# Topical rapamycin inhibits tuberous sclerosis tumor growth in a nude mouse model

**DOI:** 10.1186/1471-5945-8-1

**Published:** 2008-01-28

**Authors:** Aubrey Rauktys, Nancy Lee, Laifong Lee, Sandra L Dabora

**Affiliations:** 1 Translational Medicine Division, Department of Medicine, Brigham & Women's Hospital, One Blackfan Circle, Karp Research Building, Boston, MA, 02115 USA

## Abstract

**Background:**

Skin manifestations of Tuberous Sclerosis Complex (TSC) cause significant morbidity. The molecular mechanism underlying TSC is understood and there is evidence that systemic treatment with rapamycin or other mTOR inhibitors may be a useful approach to targeted therapy for the kidney and brain manifestations. Here we investigate topical rapamycin in a mouse model for TSC-related tumors.

**Methods:**

0.4% and 0.8% rapamycin ointments were applied to nude mice bearing subcutaneous, TSC-related tumors. Topical treatments were compared with injected rapamycin and topical vehicle. Rapamycin levels in blood and tumors were measured to assess systemic drug levels in all cohorts.

**Results:**

Treatment with topical rapamycin improved survival and reduced tumor growth. Topical rapamycin treatment resulted in systemic drug levels within the known therapeutic range and was not as effective as injected rapamycin.

**Conclusion:**

Topical rapamycin inhibits TSC-related tumor growth. These findings could lead to a novel treatment approach for facial angiofibromas and other TSC skin lesions.

## Background

Tuberous sclerosis complex (TSC) is an autosomal dominant, multi-system tumor disorder characterized by hamartomatous tumors affecting the brain, kidneys, lungs, heart and skin. Clinical manifestations of TSC were recently reviewed [1,2] and major criteria include kidney angiomyolipomas (AMLs), cardiac rhabdomyomas, facial angiofibromas, ungual or periungual fibromas, shagreeen patch, hypomelanotic macule, retinal hamartomas, subependymal nodules, subependymal giant cell astrocytomas (SEGAs), cortical tubers and lymphangioleiomyomatosis (LAM). Although TSC-associated tumors are benign, TSC patients can have a number of medical problems including epilepsy, cognitive impairment, behavior problems, brain lesions (tubers and/or subependymal nodules), skin tumors (facial angiofibromas), cardiac tumors (rhabdomyomas), kidney tumors (AMLs), kidney cysts, renal cell cancer, and pulmonary abnormalities including LAM [3-5]. The skin manifestations of TSC often lead to the diagnosis. Although there are a variety of skin manifestations, the facial angiofibromas in particular cause significant morbidity for patients because they occur on the face and current treatment options are limited [6,7].

There are two disease genes: *TSC1 *on 9q34 and *TSC2 *on 16p13 [8,9]. Their gene products, hamartin and tuberin respectively, form a tumor suppressor complex [10,11] that controls a key regulatory kinase, mammalian Target of Rapamycin (mTOR). When mutations occur in either gene, the hamartin-tuberin complex does not function properly and the mTOR pathway is constitutively activated which leads to dysregulated protein translation, cell growth and proliferation [12,13]. While a mutation in either gene has been shown to result in disease [14], *TSC2 *mutations are 5–6 times more common than *TSC1 *mutations and have been linked with a more severe phenotype [3,15,16]. As cells that lack normal tuberin or hamartin cannot down-regulate the mTOR signaling pathway, there is significant interest in investigating the utility of mTOR inhibitors, such as rapamycin and its analogs, to treat TSC-related tumors. Rapamycin (also known as sirolimus, Rapamune) is an mTOR kinase inhibitor that is FDA approved for immunosuppression following kidney transplantation. There are several rapamycin analogs (CCI-779, RAD001, and AP23575) that are under investigation as anti-tumor agents [17], and CCI-779 (also known as Temsirolimus) was recently approved for the treatment of poor risk metastatic renal cell carcinoma [18].

The beneficial effects of mTOR inhibitors have been shown in preclinical studies of TSC rodent models, where reductions were seen in kidney, subcutaneous and pituitary tumors [19-22]. Furthermore, several case reports demonstrate regression in kidney AMLs and SEGAs after rapamycin treatment [23-25] and several mTOR inhibitor trials for TSC and/or LAM are currently underway.

Skin lesions that occur in TSC include facial angiofibromas, hypomelanotic macules, shagreen patch, and ungual/periungual fibromas. Facial angiofibromas are red papules distributed across the face that begin to appear in early childhood and occur in 60–79% of patients. Hypomelanotic macules are polygonal white spots that occur in 89–97% of patients. The shagreen patch is an elevated patch or plaque on the lower back with a surface resembling an orange peel; these lesions can increase in size with age and occur in 39–51% of patients. Ungual/periungual fibromas are growths that originate from below the proximal nail fold, tend to develop in older children or adults, and occur in 15–36% of patients [3,15,16].

While TSC skin lesions are usually not life threatening, the facial angiofibromas that occur in this population are prevalent and often disfiguring, resulting in a need for improving treatment options. The current treatment options for facial angiofibromas include cryosurgery, dermabrasion, surgical excision, and laser therapy. However, effectiveness varies, complications can occur, recurrence is common, and repeated treatments are frequently necessary [2,26,27]. Here we investigate the utility of topical rapamycin as a novel therapeutic strategy for TSC skin disease by evaluating its efficacy on TSC-related tumors in a preclinical model.

## Methods

### Induction of Subcutaneous Tumors in Nude Mice and Treatment with Topical Rapamycin

Nude mice (strain CD-1nuBR, up to 6 weeks old) were obtained from Charles River Laboratories (Wilmington, Massachusetts). 64 mice were injected with 2.5 million NTC/T2null (*Tsc2-/-, Trp53-/-*) cells on their dorsal flanks as described previously [20]. Cages of 4–8 mice were randomly assigned to treatment groups before tumors appeared. As soon as tumors were visible, they were measured five days per week (Monday through Friday) using calipers. Tumor volumes were then calculated using the formula: length × width × width × 0.5 [28]. Treatment was started when tumors reached approximately 200 mm^3^. There were a total of five treatment groups: 0.8% (0.16 mg) direct topical rapamycin (n = 13), 0.8% (0.16 mg) indirect topical rapamycin (n = 12), 0.4% (0.08 mg) direct topical rapamycin (n = 15), direct topical vehicle control (n = 12), and 0.16 mg intraperitoneal (IP) rapamycin (n = 8). The direct topical treatments were applied to the skin directly over the tumor surface. The indirect topical treatment was applied to skin on the upper back, away from the tumor. All mice were treated three days a week, Monday, Wednesday and Friday. Each treatment group was divided in half with one half assigned for euthanization at 24 hours after the final treatment and the other half at 48 hours after the final treatment. Once tumors grew to 3000 mm^3^, the mice were euthanized at their assigned time point. Mice were weighed on day one of their treatment and at necropsy; no notable changes were seen in any cohorts.

Four mice were excluded from the analysis. Three of these mice (two assigned to 0.8% direct topical rapamycin and one assigned to 0.4% direct topical rapamycin) did not grow large enough tumors to reach the start volume of 200 mm^3^. One mouse (assigned to 0.8% direct topical rapamycin) was sacrificed early, when tumor volume was approximately 1100 mm^3^, due to significant weight loss from dehydration which did not appear to be treatment related. All procedures were approved by our animal institutional review board (Children's Hospital, Boston, MA) and were compliant with federal, local, and institutional guidelines on the care of experimental animals.

To prepare topical ointment, rapamycin powder was obtained from LC Laboratories (Woburn, Massachusetts) and two stocks, 40 mg/ml and 20 mg/ml, were made in ethanol and stored at -20°C. Single doses of rapamycin ointment for topical treatments were made by mixing 50 mg petroleum jelly with 10 μl of the corresponding stock in eppendorf tubes. A 0.8% ointment was made with the 40 mg/ml stock and a 0.4% ointment was made with the 20 mg/ml stock. The ointment was applied using cotton swabs immediately following mixing. Because we determined that 60% of each dose adhered to the swab or the eppendorf tube, the milligram dose of rapamycin actually applied was 0.16 mg (for the 0.8% ointment) and 0.08 mg (for the 0.4% ointment). For comparison, we also prepared rapamycin for IP injection. This was done by diluting the 20 mg/ml stock to a final concentration of 0.8 mg/ml in vehicle (5% PEG, 5% Tween-80) and using it within 24 hours. Animals treated with IP rapamcyin were injected with 0.2 ml for a rapamycin dose of 0.16 mg.

### Whole blood and tumor rapamycin levels

Whole blood and tumor rapamcyin levels were measured from all animals in the treatment study described above. Blood and tumors were harvested at 24 or 48 hours after the final treatment. Tumor samples were prepared by homogenizing 200 mg of tumor tissue in 1 ml of sterile saline. Whole blood was obtained by cardiac puncture, dispensed into an EDTA-containing blood tube, and diluted with an equal volume of sterile saline to ensure sufficient volume for rapamycin level analysis. All measured rapamycin levels were then corrected according to sample dilution. Rapamycin levels were measured by the Clinical Laboratory at Children's Hospital Boston (Boston, Massachusetts). The range of detection is 0.5 to 100 ng/ml.

To further investigate systemic rapamycin levels after topical rapamycin treatment, additional whole blood drug levels were obtained in control nude mice without tumors after single or multiple doses of rapamycin treatment. To demonstrate that significant ingestion of rapamycin ointment during grooming did not occur, Elizabethan collars (Braintree Scientific, Inc., Braintree, Massachusetts) or bandages (Tegaderm™ 6 cm × 7 cm transparent dressings, 3 M, St. Paul, Minnesota) were used in some groups of control animals (see Results). Elizabethan collars were placed on mice receiving rapamycin prior to drug administration and remained on for one hour. During this time, the mice were observed to ensure that visible absorption of rapamycin ointment had occurred. The bandages were applied to mice following treatment administration directly over the target area and remained intact for several hours in all mice (50% of bandages were intact after 24 hours and none were intact after 48 hours).

### Statistical Analyses

GraphPad Prism software (version 4.01) was used for all data analysis, with a p value ≤ 0.05 indicating statistical significance. All calculations were completed from raw data by two authors (AR and NL). A *t *test was used to test all quantitative data and the Mantel-Cox logrank analysis was used for survival data.

## Results

### Topical Rapamycin Reduces Tumor Growth and Improves Survival in Nude Mice Bearing *Tsc2*^-/- ^Tumors

To determine whether topical rapamycin treatment is a useful therapeutic approach for TSC skin disease, we investigated the efficacy of topical rapamycin using a nude mouse model for TSC-related tumors. TSC is known to be a tumor suppressor gene disorder [29,30] so the mouse model for *Tsc2*^-/- ^tumors used here is a useful generic model for TSC related tumors. It is known that the *Tsc2*^-/- ^tumors in this mouse model have constitutively activated mTOR kinase [20], and a similar defect in mTOR signaling is observed in brain and kidney tumors associated with TSC [31,32]. It is likely that activated mTOR is also present in some TSC skin lesions because loss of heterozygosity for *TSC2 *has been demonstrated in a facial angiofibroma [33] and tuberin and/or hamartin are absent in many facial angiofibromas from individuals with TSC [34].

**Figure 1:**
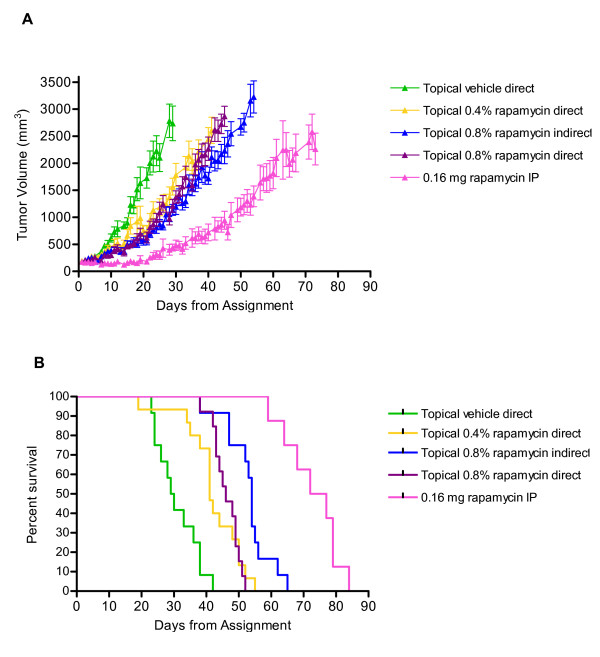
Topical rapamycin treatment impedes tumor growth and improves survival. Average tumor growth (A) and survival curves (B) for indicated treatment groups.

A cohort of 64 nude mice was injected with NTC/T2null cells. This cohort was divided into 5 treatment groups: 0.8% direct, 0.8% indirect (shoulder), 0.4% direct, 0.16 mg IP, and topical vehicle control. Doses were based on pilot studies, Ormerod et al. 2005, and drug level studies [35]. Animals began treatment when their tumor volume reached ~200 mm^3 ^and were euthanized at a tumor volume of ~3000 mm^3^. Four animals were excluded from analyses as previously described in the methods. Average tumor growth is shown for each treatment group in Figure 1A. The data points shown represent days when greater than or equal to half of the group was treated and had tumors measured. On day 29, the average tumor volumes for the 0.4% direct (1568 ± 155 mm^3^), 0.8% direct (1212 ± 118 mm^3^), and 0.8% indirect (1136 ± 101 mm^3^) were all significantly lower than the vehicle-treated cohort (2736 ± 321 mm^3^) (Table 1). Improved survival was also seen in all treated cohorts when compared to the vehicle-treated cohort (Table 1, Figure 1B). Although administering the identical rapamycin dose by IP injection is more effective, this study demonstrates that rapamycin applied topically does impede TSC tumor growth when compared to the vehicle.

**Table 1:** Survival and Tumor Growth Analysis

	0.4% Direct Topical Rapamycin	0.8% Indirect Topical Rapamycin	0.8% Direct Topical Rapamycin	0.16 mg Injected (IP) Rapamycin	Direct Topical Vehicle
Median Survival (days)	41	54	46	74.5	29.5
Logrank Analysis*	*p = 0.0003*	*p =< 0.0001*	*p =< 0.0001*	*p =< 0.0001*	N/A
Logrank Analysis**	p = 0.62	*p = 0.0003*	N/A	*p =< 0.0001*	N/A
					
Ave. Tumor Vol. (mm^3^)					
Day 29	1568 ± 155	1136 ± 101	1212 ± 118	440 ± 65	2736 ± 321
T test*	*p = 0.0014*	*p =< 0.0001*	*p =< 0.0001*	*p =< 0.0001*	N/A
T test**	p = 0.08	p = 0.63	N/A	*p = 0.0001*	N/A
Day 45	N/A	2235 ± 246	2869 ± 188	944 ± 166	N/A
T test**	N/A	p = 0.06	N/A	*p =< 0.0001*	N/A
					
Number of Mice	15	12	13	8	12

*Compared to vehicle.

**Compared to 0.8% direct topical rapamycin

### Comparison of Direct and Indirect Treatment

To test whether topical rapamycin applied to skin directly over the subcutaneous tumor was more effective than a topical dose applied several centimeters away from the tumor surface, the two 0.8% groups were compared. When evaluating survival between these groups, the difference is significant and to our surprise indicated that the indirect topical treatment was more effective than the direct treatment (Table 1). On day 45, the difference in average tumor volumes for the two groups did not meet our criteria for statistical significance (direct topical rapamycin 1568 ± 155 mm^3^, indirect topical rapamycin 1212 ± 118 mm^3^), but the p value was 0.06.

### Rapamycin Drug Level Measurements show Systemic Levels are Achieved through Topical Absorption

**Figure 2:**
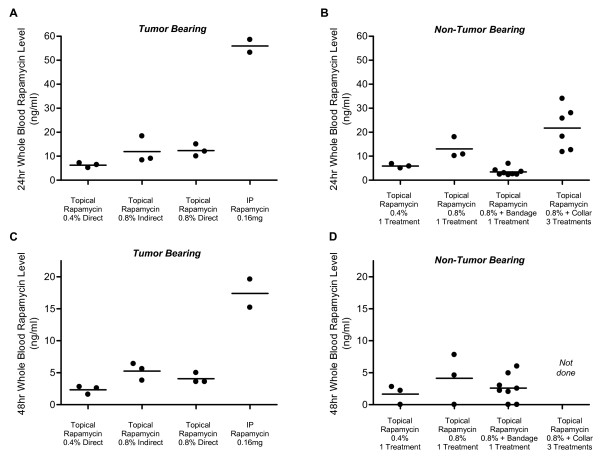
Rapamycin whole blood levels after treatment with topical and IP rapamycin. A) Whole blood rapamycin levels from tumor bearing animals from indicated treatment groups. Rapamycin levels were measured 24 hours after the final dose of rapamycin. B) Whole blood rapamycin levels were measured in cohorts of control mice with no tumors. As indicated, levels were measured 24 hours after either one or three doses of topical rapamycin. In some groups, Elizabethan collars or bandages were used to prevent ingestion of rapamycin ointment. All animals had rapamycin levels >3 ng/ml. The slightly higher levels in the Elizabethan collar group suggests that ingestion is not a major issue. The lower levels in the bandage groups suggests that the bandage polymer can affect drug absorption. C) Whole blood rapamycin levels were also measured 48 hours after the final dose in tumor bearing animals. D) In three of the control, non-tumor bearing groups, rapamycin levels were measured at 48 hours following their final treatment to compare to tumor bearing animals.

**Figure 3:**
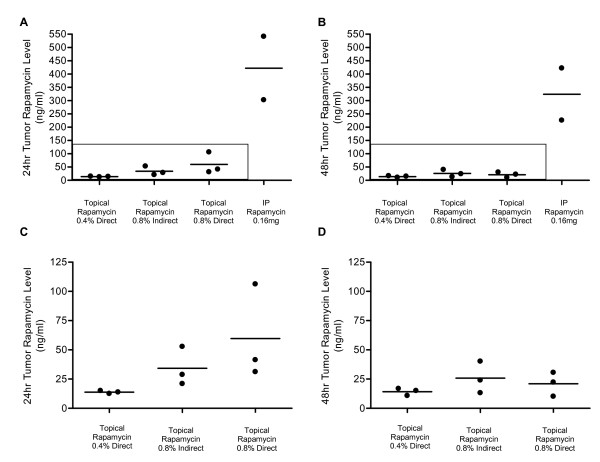
Rapamycin levels in tumor samples after treatment with topical or IP rapamycin. Rapamycin levels in tumor homogenates from indicated treatment groups were measured 24 hours (A, C) and 48 hours (B, D) after the final dose of rapamycin. Panels C and D are enlarged versions of the boxed-in data in panels A and B respectively.

Rapamycin levels were measured in whole blood (Figure 2, Table 2) and tumors (Figure 3, Table 3) from a subset of the cohort described above. For whole blood levels, rapamycin level measurements were taken 24 and 48 hours after the last dose of drug (Figure 2A and 2C). As demonstrated, levels in the 0.8% topical groups (direct and indirect) are comparable to levels in corresponding non-tumor bearing animals (Figure 2B and 2D). This is true for the 0.4% dose as well. When a 0.16 mg dose is given by injection, the 24 hour levels in whole blood are ~5 times higher than when administered topically. Rapamycin levels in tumors are shown in Figure 3 and Table 3. Both 24 hour and 48 hour levels are shown for all treatment groups. Both 0.8% direct and indirect topical doses resulted in similar levels and were ~2–4 times higher than the 0.4% direct topical dose. Although the direct application gave a higher average tumor drug level at 24 hours, this difference was not statistically significant and was inconsistent with the better survival observed with indirect treatment. We are unable to explain this inconsistency and conclude that overall, the two methods gave very similar results. This data demonstrates that therapeutic systemic levels of rapamycin were achieved after topical administration in these animals.

**Table 2:** Whole Blood Rapamycin Levels

	24 hours			48 hours		
	
**Treatment Groups**	Rapamycin Level (ng/ml)	p value*	Number of Mice	Rapamycin Level (ng/ml)	p value**	Number of Mice
Treatment Trial (*Tumor bearing*)						
0.4% Direct Topical Rapamycin	6.3 ± 0.6	*0.02*	3	2.3 ± 0.4	*0.04*	3
0.8% Indirect Topical Rapamycin	11.9 ± 3.2	0.92	3	5.3 ± 0.8	0.25	3
0.8% Direct Topical Rapamycin	12.3 ± 1.5	N/A	3	4.1 ± 0.5	N/A	3
0.16 mg Rapamycin IP	55.9 ± 2.7	*0.0005*	2	17.4 ± 2.2	*0.005*	2
						
*Non-Tumor bearing*						
0.4% Topical Rapamycin (1 dose)	5.9 ± 0.5	*0.01*	3	1.7 ± 0.9	0.07	3
0.8% Topical Rapamycin (1 dose)	13.0 ± 2.5	0.81	3	4.1 ± 2.3	0.98	3
0.8% Topical Rapamycin + Bandage (1 dose)	3.4 ± 0.6	*<0.0001*	8	2.6 ± 0.7	0.28	8
0.8% Topical Rapamycin + Collar (3 doses)	21.7 ± 3.7	0.13	6	Not done	N/A	Not done

* Compared to 0.8% direct treatment (tumor bearing) 24 hr blood level.

** Compared to 0.8% direct treatment (tumor bearing) 48 hr blood level

**Table 3:** Tumor Rapamycin Levels

	24 hours			48 hours		
	
**Treatment Groups**	Rapamycin Level (ng/ml)	p value*	Number of Mice	Rapamycin Level (ng/ml)	p value**	Number of Mice
Treatment Trial (*Tumor bearing*)						
0.4% Direct Topical Rapamycin	13.8 ± 0.7	0.12	3	14.2 ± 1.8	0.33	3
0.8% Indirect Topical Rapamycin	34.2 ± 9.6	0.37	3	25.8 ± 7.8	0.65	3
0.8% Direct Topical Rapamycin	59.6 ± 23.5	N/A	3	21.0 ± 5.9	N/A	3
0.16 mg Rapamycin IP	421.8 ± 119.4	*0.03*	2	323.7 ± 98.1	*0.03*	2

* Compared to 0.8% direct treatment 24 hr tumor level.

** Compared to 0.8% direct treatment 48 hr tumor leve

In order to verify that the topical rapamycin dose used in these experiments is relevant to the effective dose approved for use in humans, we measured whole blood rapamycin levels in non-tumor bearing animals after single and multiple doses. The rapamycin level in whole blood 24 hours after a single 0.8% dose of topical rapamycin was 13.0 ± 2.5 ng/ml. After a single 0.4% dose the rapamycin level was 5.9 ± 0.5 ng/ml. These 24 hour levels are within the typical therapeutic range for rapamycin when used for immunosuppression after organ transplantation (3–15 ng/ml). 48 hour levels were also measured and show that in animals treated with a single 0.4% dose, rapamycin levels dropped below therapeutic range (1.7 ± 0.9 ng/ml) while those treated with a single 0.8% dose did not (4.1 ± 2.3 ng/ml). In addition, we measured rapamycin levels using two barrier methods to ensure that ingestion due to grooming was not a contributing factor (Figure 2B and 2D, Table 2). Several animals were fitted with Elizabethan collars for one hour after topical rapamycin was applied so they were unable to groom the area with ointment. There were 6 animals in the Elizabethan collar group and all were treated with three 0.8% doses of topical rapamycin (on days 1, 4 and 6) with the average rapamycin level in whole blood at 24 hours after the last dose being 21.7 ± 3.7 ng/ml. As this level was slightly higher but not significantly different than in animals with no barriers to prevent grooming, it provides evidence that grooming does not seem to increase whole blood levels of rapamycin after topical treatment. We also used bandages (Tegaderm™ 6 cm × 7 cm transparent dressings) as a grooming prevention strategy and were surprised to find significantly lower rapamycin levels in this group at 24 hours but not 48 hours. This suggests that the bandages may significantly alter absorption kinetics of rapamycin so that the dose penetrating the skin is lower at 24 hours. Our finding that the 48 hour level in the bandage group is not significantly different from the 48 hour levels in the no barrier topical group suggests that the bandage may provide a means for sustained release of topical rapamycin, but this will require further studies. In summary, these data demonstrate that a relevant dose of topical rapamycin was used, it is unlikely that drug ingestion during grooming increases drug levels, and Tegaderm™ bandages may affect rapamycin ointment absorption as applied for these studies.

## Discussion

We have shown through survival and tumor growth analyses that rapamycin administered topically is an effective treatment for reducing TSC tumor growth in a preclinical model. The efficacy of topical rapamycin has not previously been studied in TSC preclinical models, but prior studies have shown that 0.4–3.6% topical rapamycin was effective in a mouse model for irritant dermatitis [36] and 1% topical rapamycin was effective in a mouse model of allergic dermatitis [37]. In a randomized, double-blind clinical trial, 8% topical rapamycin was effective for the treatment of psoriasis and there was evidence of skin penetration without measurable rapamycin in blood [35].

There is also evidence of a reduced incidence of skin and non-skin cancers when rapamycin is used for immunosuppression instead of other agents following kidney transplantation [38,39]. Furthermore, systemic rapamycin treatment is effective in the treatment of Kaposi's Sarcoma [40,41]and in preclinical studies of melanoma [42]. Finally, a study showing decreased expression of the *TSC2 *gene product, tuberin, in sporadic squamous and basal cell carcinomas suggests that mTOR inhibitors may be useful in treating these common skin cancers as well [43].

In our studies of topical rapamycin for TSC tumors, we found that there was no advantage to applying topical rapamycin directly to the tumor surface compared with indirect topical application. In fact, the indirect treatment was slightly more effective despite similar drug levels in tumors and whole blood from these cohorts. We also note that a limitation of this study is that mice have a significantly higher ratio of surface area:volume compared with humans so both the indirect and direct topical treatments resulted in 24 hour whole blood and tumor rapamycin levels within the known therapeutic range for effective immunosuppression in humans. It is therefore likely that the effect of topical rapamycin in these experiments was due to systemic rapamycin exposure. Despite this limitation, this study demonstrates that 0.4–0.8% rapamycin applied topically does penetrate the skin in this preclinical model.

## Conclusion

In summary, we have shown that topical administration of rapamycin is an effective treatment for TSC-related tumors in a mouse model. Our data demonstrates that transdermal delivery of rapamycin is feasible and topical rapamycin should be further investigated as a novel treatment approach for TSC skin disease such as facial angiofibromas. Furthermore, the utility of topical rapamycin should be further investigated for other skin disorders such as psoriasis, Kaposi's Sarcoma, basal cell carcinoma, and squamous cell carcinoma.

## List of Abbreviations

TSC – Tuberous Sclerosis Complex; mTOR – Mammalian target of rapamycin; ng – nanogram; mg – milligram; ml – milliliter; mm – millimeter; cm – centimeter; PEG – Polyethylene glycol; AML – Angiomyolipoma; SEGA – Subependymal giant cell astrocytoma; LAM – Lymphangioleiomyomatosis; IP – intraperitoneal

## Competing interests

The author(s) declare that they have no competing interests.

## Authors' contributions

SD provided funding, critical guidance for the experiments, and was responsible for supervising the writing and editing of the manuscript. AR designed most of the experiments, collected most of the data, performed all statistical analysis, and wrote the first draft of the manuscript. NL assisted with experimental design, data collection, and independent verification of statistical analyses. LL assisted in planning the experiments, critical evaluation of data, and editing the manuscript. All authors have read and approved the final manuscript.

## Pre-publication history

The pre-publication history for this paper can be accessed here:


